# Protein adsorption/desorption dynamics on Ca-enriched titanium surfaces: biological implications

**DOI:** 10.1007/s00775-021-01886-4

**Published:** 2021-08-27

**Authors:** Francisco Romero-Gavilán, Andreia Cerqueira, Eduardo Anitua, Ricardo Tejero, Iñaki García-Arnáez, Cristina Martinez-Ramos, Seda Ozturan, Raul Izquierdo, Mikel Azkargorta, Félix Elortza, Mariló Gurruchaga, Isabel Goñi, Julio Suay

**Affiliations:** 1 Department of Industrial Systems Engineering and Design, Universitat Jaume I, Campus del Ríu Sec, Av. Vicent Sos Baynat s/n, 12071 Castellón de la Plana, Spain; 2 BTI Biotechnology Institute I+D, C/ Leonardo da Vinci 14B, 01510 Miñano, Spain; 3 University Institute of Regenerative Medicine and Oral Implantology (UIRMI), University of the Basque Country (UPV/EHU), C/ Jacinto Quincoces, 39, 01007 Vitoria, Spain; 4 Private Practice in Oral Implantology, C/Jose Maria Cagigal, 19, 01007 Vitoria, Spain; 5 Facultad de Ciencias Químicas, Universidad del País Vasco, P. M. de Lardizábal, 3, 20018 San Sebastián, Spain; 6 Center for Biomaterials and Tissue Engineering, Universitat Politècnica de Valencia, Camino de Vera, s/n, 46022 Valencia, Spain; 7 Department of Periodontology, Faculty of Dentistry, Istanbul Medeniyet University, Istanbul, Turkey; 8 Proteomics Platform, CIBERehd, ProteoRed-ISCIII, CIC bioGUNE, Basque Research and Technology Alliance (BRTA), Bizkaia Science and Technology Park, 48160 Derio, Spain

**Keywords:** Proteomics, Bioinorganic chemistry, Dental implants, Osseointegration, Blood clotting

## Abstract

Calcium ions are used in the development of biomaterials for the promotion of coagulation, bone regeneration, and implant osseointegration. Upon implantation, the time-dependent release of calcium ions from titanium implant surfaces modifies the physicochemical characteristics at the implant–tissue interface and thus, the biological responses. The aim of this study is to examine how the dynamics of protein adsorption on these surfaces change over time. Titanium discs with and without Ca were incubated with human serum for 2 min, 180 min, and 960 min. The layer of proteins attached to the surface was characterised using nLC-MS/MS. The adsorption kinetics was different between materials, revealing an increased adsorption of proteins associated with coagulation and immune responses prior to Ca release. Implant–blood contact experiments confirmed the strong coagulatory effect for Ca surfaces. We employed primary human alveolar osteoblasts and THP-1 monocytes to study the osteogenic and inflammatory responses. In agreement with the proteomic results, Ca-enriched surfaces showed a significant initial inflammation that disappeared once the calcium was released. The distinct protein adsorption/desorption dynamics found in this work demonstrated to be useful to explain the differential biological responses between the titanium and Ca-ion modified implant surfaces.

**Graphic abstract:**

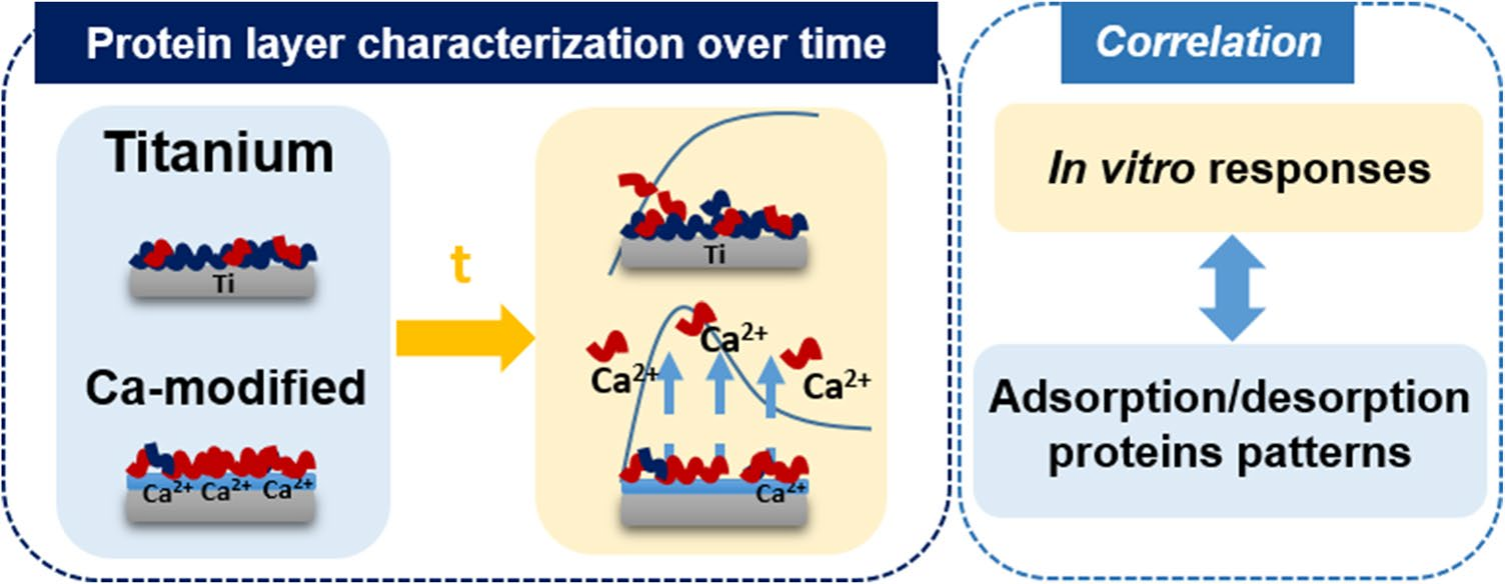

**Supplementary Information:**

The online version contains supplementary material available at 10.1007/s00775-021-01886-4.

## Introduction

Bivalent cationic ions, such as calcium, have been studied as key factors to improve the bone regeneration process around biomaterials [1, 2]*.* Calcium, one of the major components of biological apatite (Ca_10_(PO_4_)_6_(OH)_2_), is the most abundant metal element in the human body, playing an essential role in bone metabolism and blood coagulation [3]*.* The platelets are pivotal in the processes of haemostasis and blood clotting, which precede tissue repair. Calcium mediates the binding of the platelet membrane phospholipids to factor Xa and factor IXa, which are required for the operation of the tenase and prothrombinase complexes. These complexes convert prothrombin into thrombin (factor IIa), which triggers fibrin polymerisation [4]. The characteristics of the resulting clot will condition the subsequent regeneration process. Additionally, Ca^2+^ can favour osteoblast proliferation, mineralization, and extracellular matrix mineralization [5]. This key role of calcium in bone tissue regeneration has turned this element into an interesting option in the development of biomaterials [6]. In the field of implant dentistry, calcium was used to bioactivate the implant surfaces and enhance their osseointegration [7–10]. Doe et al. [9] found that acid-etched pure titanium implants with calcium ion surface modification showed remarkably high osteogenic activity and high stability in osseous tissue. On the other hand, Anitua et al. [10] evaluated titanium implant surfaces modified with CaCl_2_, showing that these surfaces were able to stimulate osteoblastic cell attachment, proliferation, and differentiation, as well as improved implant osseointegration in vivo. Calcium ions on TiO_2_ surfaces can activate platelets and the exocytosis of the alpha and dense granules [11]*.* Additionally, calcium on the implant surface could enhance the rate of protein adsorption, acting as a protein-binding site and conditioning the type of protein that adheres to the implant [12].

Protein adsorption onto a biomaterial surface is the first event to occur after implantation, playing a crucial role in determining the consequent biological responses and the material biocompatibility [13]. In most studies of protein adsorption, only single or very few serum proteins have been examined [14, 15], providing valuable insights into the biological responses to biomaterials. However, it is not clear whether such results can be used to understand competitive adsorption from mixtures [16]. Looking into simultaneous adsorption of proteins onto the same surface would be preferable since it is closer to the reality experienced by the biomaterial in the biological environment. The study of competitive adsorption of proteins is essential, as biomaterials usually are exposed to complex protein mixtures (e.g., from blood plasma) with different affinities for the surfaces [17, 18]. During single protein adsorption experiments, the maximum concentration of a given protein on the surface is easily achieved. On the other hand, in complex protein mixtures, these molecules diffuse to the surface at different rates. At first, the high-mobility or more concentrated proteins will be adsorbed; however, over time, they will be displaced by other proteins with lower concentration or larger molecular weight, but higher affinity to the surface. This phenomenon is commonly referred to as the “Vroman effect” [16, 19]. Although this competitive displacement has been intensively researched, it is still not well understood [19]. Recently, an increasing number of proteomic studies have examined simultaneous adsorption of multiple proteins on biomaterial surfaces [8–11]. Mass spectrometry-based proteomics studies have demonstrated to be a powerful approach to analyse proteins on a large scale [20]. The composition and conformation of the protein layer adsorbed at this interface will determine the nature of the reciprocal tissue–material fate. The coagulation and complement cascades can be activated by proteins attached to the material, resulting in the onset of inflammation [21] and shaping the crucial characteristics of cellular response, including adhesion, spreading, migration, proliferation and differentiation [22, 23]. In turn, the surface properties of the materials condition the adsorption of proteins. The extent and the manner of protein adsorption on surfaces can be significantly affected by the local environment and surface properties such as wettability, roughness, surface charge, surface chemistry, the concentration of ions and temperature [24]. Additionally, although it has not yet been studied in-depth, changes in the biomaterial surface due to its interaction with the biological environment could alter the protein layer properties over time.

The current study aimed to analyse and compare the adsorption movements of human serum proteins over time on both a titanium surface and the same Ti surface modified with CaCl_2_ through proteomic methods. Additionally, the osteogenic and inflammatory potential of these surfaces were studied in vitro employing primary human alveolar osteoblasts and THP-1 monocytes cell lines, respectively. The changes in the protein patterns were correlated with the in vitro cellular responses.

## Materials and methods

### Sample preparation

Three types of samples machined from CP titanium grade IV were prepared: discs of 6-mm diameter and 1-mm thickness were used for osteoblast adhesion and proliferation tests and proteomics; discs of 12.7-mm diameter and 1-mm thickness were used to carry out ELISA assays; and implants Interna^®^ Core^®^ 4.25 × 10 mm (BTI Biotechnology Institute S.L., Vitoria, Spain) were employed for implant-blood contact experiments. All the samples were subjected to the same proprietary process of roughening by sequential acid etching and further cleaning and conditioning in a cleanroom class A (BTI). Acid-etched samples without further modifications were beta-ray sterilised and stored until use (control). Calcium ion-modified surfaces (Ca) were prepared following a proprietary process (BTI). Briefly, the discs were dip-coated in a 5 wt% CaCl_2_ solution after roughening and before sterilisation. For in vitro testing, Ca surfaces were sonicated (3 × 3 min) in Nanopure water obtained with a Milli-Q Direct water system (Millipore, Madrid, Spain), and then the water was blown off with a stream of filtered nitrogen before analysis to simulate the state of complete Ca release and thus, understanding the biological response of this treatment after the release of this cation (Ca-released).

### Physicochemical characterization

Scanning electron microscopy (SEM) with a Leica-Zeiss LEO equipment (Leica, Wetzlar, Germany) coupled with energy-dispersive X-ray spectroscopy (EDX, Leica-Zeiss LEO) was used to analyse the morphology and chemistry of the studied surfaces. Platinum sputtering was used to increase the conductivity of the samples for the SEM examination.

### Protein adsorption kinetics

#### Protein layer formation

Control and Ca discs were incubated for 2 min, 180 min, and 960 min with 1 mL of human blood serum from male AB plasma (Merck, Darmstadt, Germany) in a 24-well plate in a humidified atmosphere (37 °C, 5% CO_2_). Then, the serum was removed, and the discs were rinsed five times with ddH_2_O and once with 100 mM NaCl (Merck), 50 mM Tris–HCl (Merck), pH 7.0, to eliminate the non-adsorbed proteins. The adsorbed protein layer was eluted by washing the discs in 0.5 M triethylammonium bicarbonate buffer (TEAB; Merck) with 4% sodium dodecyl sulphate (Merck) and 100 mM of dithiothreitol (Merck). Four independent experiments were carried out for each type of surface and incubation time; in each experiment, four discs for each material were processed. The serum protein content was quantified before the experiment (Pierce BCA assay kit; Thermo Fisher Scientific, NY, USA), obtaining a value of 45 mg mL^−1^.

#### Proteomic analysis

Proteomic analysis was carried out as described by Romero-Gavilán et al*.* [25], with minor variations. Briefly, the eluted protein was digested in-solution, following the FASP protocol established by Wisnewski et al. [26]. The obtained peptides were resuspended in 0.1% formic acid. The samples were analysed with nano-scale liquid chromatographic tandem mass spectrometry (nLC-MS/MS) by loading them onto a nanoACQUITY UPLC system connected online to an LTQ Orbitrap XL ETD (Thermo Electron, Bremen, Germany). Each material was analysed in quadruplicate. Progenesis software (Nonlinear Dynamics, Newcastle, UK) was employed to perform differential protein analysis as described before [25]. The protein classification by functions was carried out using the DAVID Go annotation programme (https://David.ncifcrf.gov/) and PANTHER classification system (http://www.pantherdb.org/).

### Implant–blood contact experiments

Whole blood from one young healthy donor was collected in 3.8% sodium citrate (w/v) containing tubes (BTI Biotechnology Institute S.L.) after informed consent and stored at 37 °C until testing. Nine implants (4.25 × 10 mm), 3 per surface type, were introduced vertically and apically into glass wells filled with blood until at least the last thread of the implant was covered with blood. After 20 min, the implants were pulled out. Photos were taken with a Canon EOS 5D Mark IV equipped with an EF 100 mm f/2.8L Macro IS USM objective (Canon Inc., Tokyo, Japan) upon first implant contact with blood and at the extraction of the implants.

### In vitro studies

Control and Ca-modified surface discs were tested in vitro with human osteoblasts and monocytes to evaluate osteogenic and inflammatory responses, respectively. As Ca-modified surfaces change over time, they were studied in two stages: in the initial phase (Ca), which represents the original surface that interacts with the blood after implantation; and the second one, which represents the treatment once the Ca is released (Ca-released).

#### Osteoblast cell culture

Primary human alveolar osteoblasts were isolated as previously described [27] and cultured in the osteoblast basal medium (ObM; ScienCell Research Laboratories, Carlsbad, CA, USA) supplemented with 50 µg/mL gentamicin (ScienCell Research Laboratories) and 15% foetal bovine serum (FBS; Biochrom, Berlin, Germany). Osteoblast cultures were maintained at 37 °C in a humidified 5% CO_2_ atmosphere. Cells between passages 4 and 6 were used for experiments. Two independent experiments for each assay were carried out. In each experiment, the surfaces were tested in triplicate.

#### Osteoblast adhesion and viability

Six-mm diameter discs were placed in 96-well optical bottom plates (Thermo Fisher Scientific). Polystyrene surfaces of the 96-well plates were used as a positive control. Osteoblasts were seeded at a density of 2 × 10^4^ cells/cm^2^ and allowed to adhere. After 60 or 90 min, the culture medium was discarded, the adhered cells were rinsed with phosphate-buffered saline (PBS) and frozen at − 80 °C. DNA from attached cells was quantified using CYQUANT cell proliferation assay (Molecular Probes, Invitrogen, Grand Island, NY), following the manufacturer’s instructions. For the viability assays, the osteoblasts were seeded at 8 × 10^3^ cells/cm^2^. After 72 h, the protocol for DNA quantification was followed.

#### Synthesis of extracellular matrix proteins

Discs of 12.7-mm diameter with the different surface treatments were placed in 24-well plates (Thermo Fisher Scientific). The osteoblasts were seeded at the density of 8 × 10^3^ cells/cm^2^. Cells incubated on an empty well were used as control. After 3 days, the culture medium was refreshed. After 7 days of culture, the medium was aspired and stored frozen (− 80 °C) until further analysis. Quantification of the osteocalcin and procollagen type I was performed using ELISA kits (Takara, Shiga, Japan), following the manufacturer’s instructions.

#### Monocyte/macrophage cell culture

The human monocytic cell line (THP-1; ECACC 88081201) was cultured in Roswell Park Memorial Institute (RPMI) 1640 medium (Gibco, Thermo Fisher Scientific) supplemented with 10% FBS (Gibco) and 1% penicillin/streptomycin (Gibco), in a cell culture incubator (37 °C, 5% CO_2_ and 95% relative humidity). To activate the differentiation of THP-1 cells into macrophages, 50 ng/mL of phorbol-12-myristate-13-acetate (PMA) was added to the culture medium. Cells were seeded onto the discs (12.7-mm diameter) with the three distinct surfaces (30 × 10^4^ cells/cm^2^) in quadruplicate (*n* = 4) and cultured for 1 and 3 days.

#### Cytokine secretion measurements

The secretion of tumour necrosis factor α (TNF-α) and transforming growth factor-β (TGF-β) to the cell culture media was examined. After 1 and 3 days of culture, the medium was aspired and stored frozen (− 80 °C) until further analysis. The concentrations of TNF-α (pro-inflammatory) and TGF-β (anti-inflammatory) were determined using ELISA kits (Thermo Fisher Scientific) according to the manufacturer’s instructions.

### Statistical analysis

Physicochemical characterization and in vitro assay data were submitted to one-way analysis of variance (ANOVA) and a Newman–Keuls multiple comparison post-hoc test. Proteomic data were submitted to Student’s *t* test. The differences were considered statistically significant at *p* ≤ 0.05 (*), *p* ≤ 0.01 (**), and *p* ≤ 0.001 (***). Proteins were considered as differentially adsorbed when they showed statistically significant differences in the normalized abundance and a normalized abundance ratio between surfaces higher than 1.5 in either direction.

## Results

### Surface characterization

**Fig. 1:**
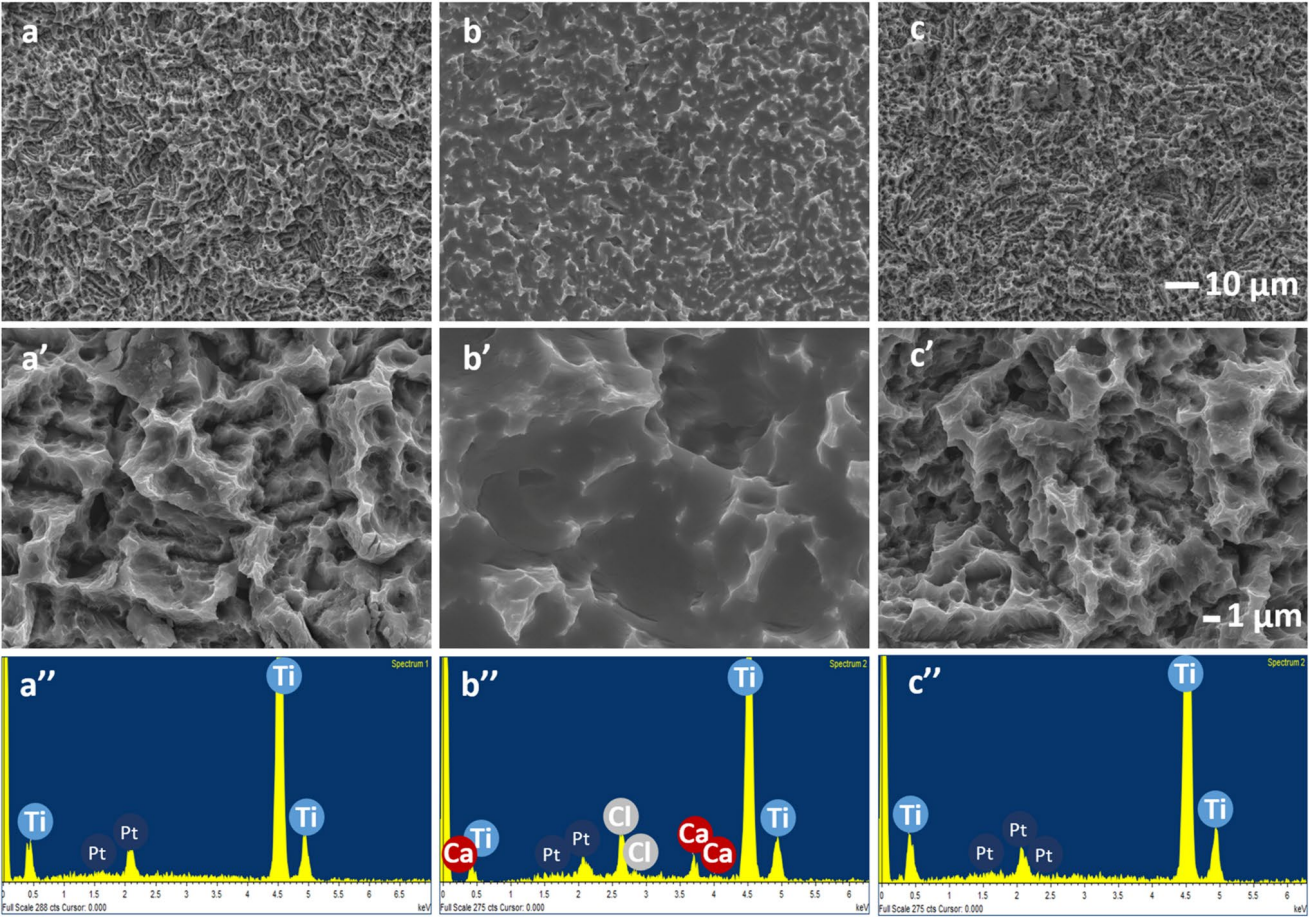
SEM micrographs of the surfaces: **a** control, **b** Ca and **c** Ca-released. Magnifications: **a**–**c** × 1000 and **a′**–**c′** × 5000. EDX chemical analysis of **a″** control, **b″** Ca and **c″** Ca-released surfaces

Figure 1 shows SEM micrographs and EDX analysis. Figure 1b–b′ shows that the obtained CaCl_2_ modification was homogeneously interspersed on the titanium surface (Ca). The CaCl_2_ treatment fills partly the cavities present at the unmodified titanium (control; Fig. 1b–b′). After washing (Ca-released), the aspect of the surface returns to the morphological characteristics prior to the Ca modification (Fig. 1c-c′). EDX of Ca samples showed peaks of calcium and chlorine in addition to the titanium substrate peaks that were observed at the Control (Fig. 1a″–b″). No presence of CaCl_2_ was found in the Ca samples after washing (Fig. 1c″).

### Proteomic analysis

Online Resource 1 shows the results of Progenesis comparative analysis. Significant differences were observed between the patterns of proteins adsorbed onto Ca and Control surfaces, for each incubation period. A total of 19 proteins were identified as differentially adsorbed onto the tested surfaces. After 2 min of incubation with the serum, seven proteins were significantly more attached to calcium-enriched samples than to Ti control surfaces. Three were related to the coagulation cascade: FA10 (41.24-fold), THRB (2.52-fold), and ANT3 (2.23-fold), and one related to an inhibitory activity of the calcium channel (AMBP). The remaining three were associated with the immune system: complement proteins C1S (2.98-fold), CO9 (2.52-fold), and the immunoglobulin KV302.

After 180 min of incubation, six proteins were significantly more abundant on calcium-containing surfaces. Two of these were related to the coagulation cascade: FA10 (130.24-fold) and THRB (3.02-fold) and one to selenium binding: SEPP1 (4.96-fold). Three more were related to the immune system: C1R (3.34-fold), CO4A (2.95-fold), and immunoglobulin IGJ. SAMP, a glycoprotein related to calcium ion binding and the innate immune response, was significantly less attached to the Ca samples in comparison with Control (12.5-fold).

Finally, after 960 min of incubation with the serum, ten proteins were found differentially attached to Ca samples in comparison with the reference. Only one protein showed an increase in its affinity to the Ca surfaces: FA10 (19.73-fold). The remaining nine proteins were significantly less attached to these surfaces. Two proteins were related to coagulation, FA12 (3.23-fold) and ANT3. Five were related to the immune system: the immunoglobulins IGHG3 (2.04-fold), KV121, IGHM and IGHG1; and the glycoprotein SAMP (5.00-fold). Two other proteins showing reduced binding to the Ca surfaces were, CERU (3.13-fold; related to copper transport) and APOB (4.35-fold), responsible for the transport of lipid molecules.

**Fig. 2:**
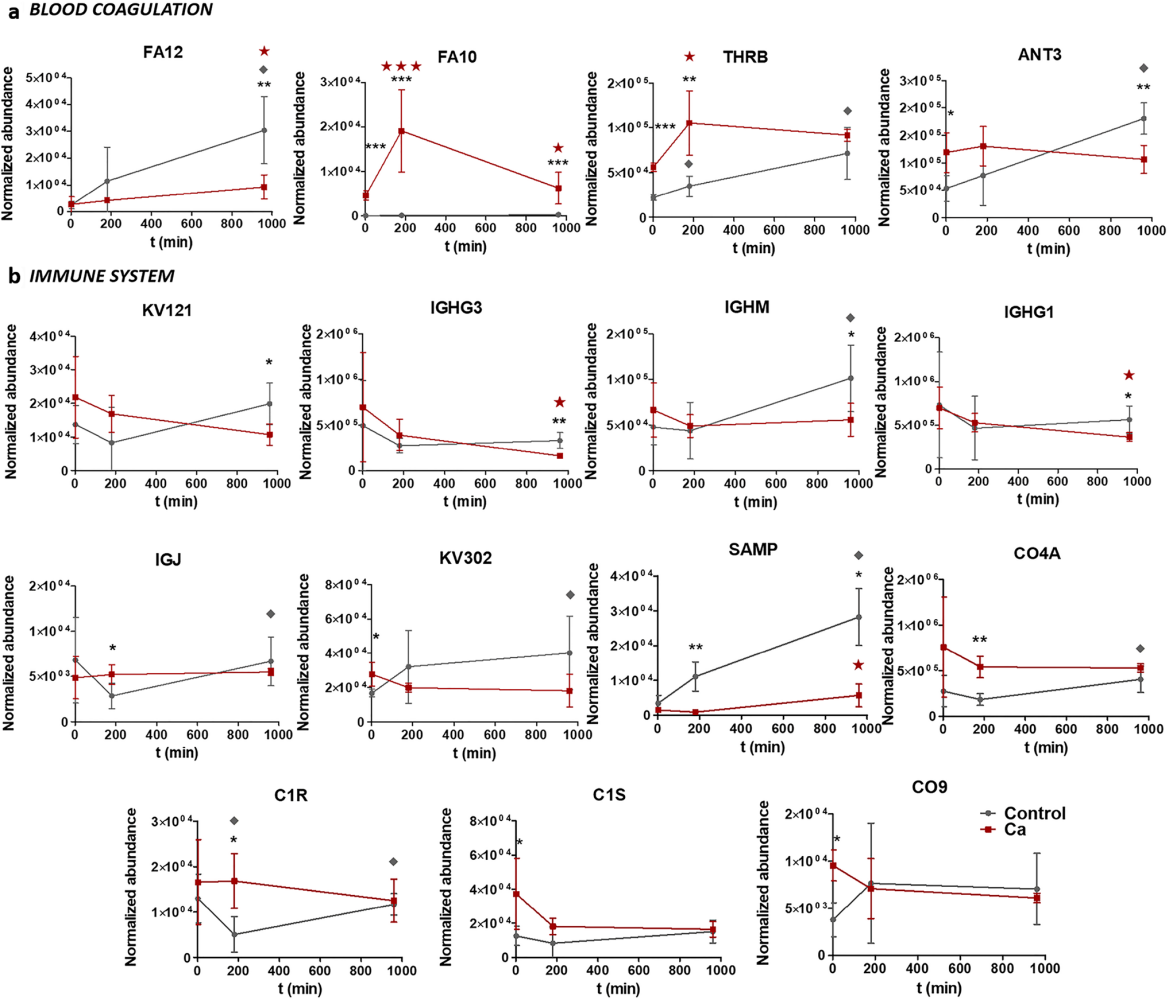
Normalised abundance of the differential proteins related to functions in **a** blood coagulation and **b** immune system after 2 min, 180 min and 960 min of incubation with human serum. Results are shown as mean ± SE. The asterisks [*p* ≤ 0.05 (*), *p* ≤ 0.01 (**) and *p* ≤ 0.001 (***)] indicate statistically significant differences in the adsorption of the protein between Control and Ca surfaces. Red stars (Ca) and grey diamonds (control) show statistical differences in protein adsorption between consecutive times of testing

Figure 2 shows the adsorption kinetics of coagulation- and immune-related proteins after different periods of contact with human serum for the Ca and Control samples. The plots present the normalised abundance of the 15 differential proteins, which were related to functions in these systems. In addition to the differences in the amount of specific proteins adhered to each of the tested surfaces, the adsorption kinetics of these proteins also showed distinct trends in each material. In general, the adsorption of most differential proteins increases over time for Control samples. Regarding the Ca surface, THRB, FA12 and SAMP increase their adhesion onto this surface over time. However, the immunoglobulins IGHG1 and IGHG3 reduce their affinity for the Ca samples. Interestingly, the FA10 protein increases its adsorption on this material during the first 180 min of assay; then, a process of desorption is detected and its abundance in the formed protein layer decreases.

**Fig. 3:**
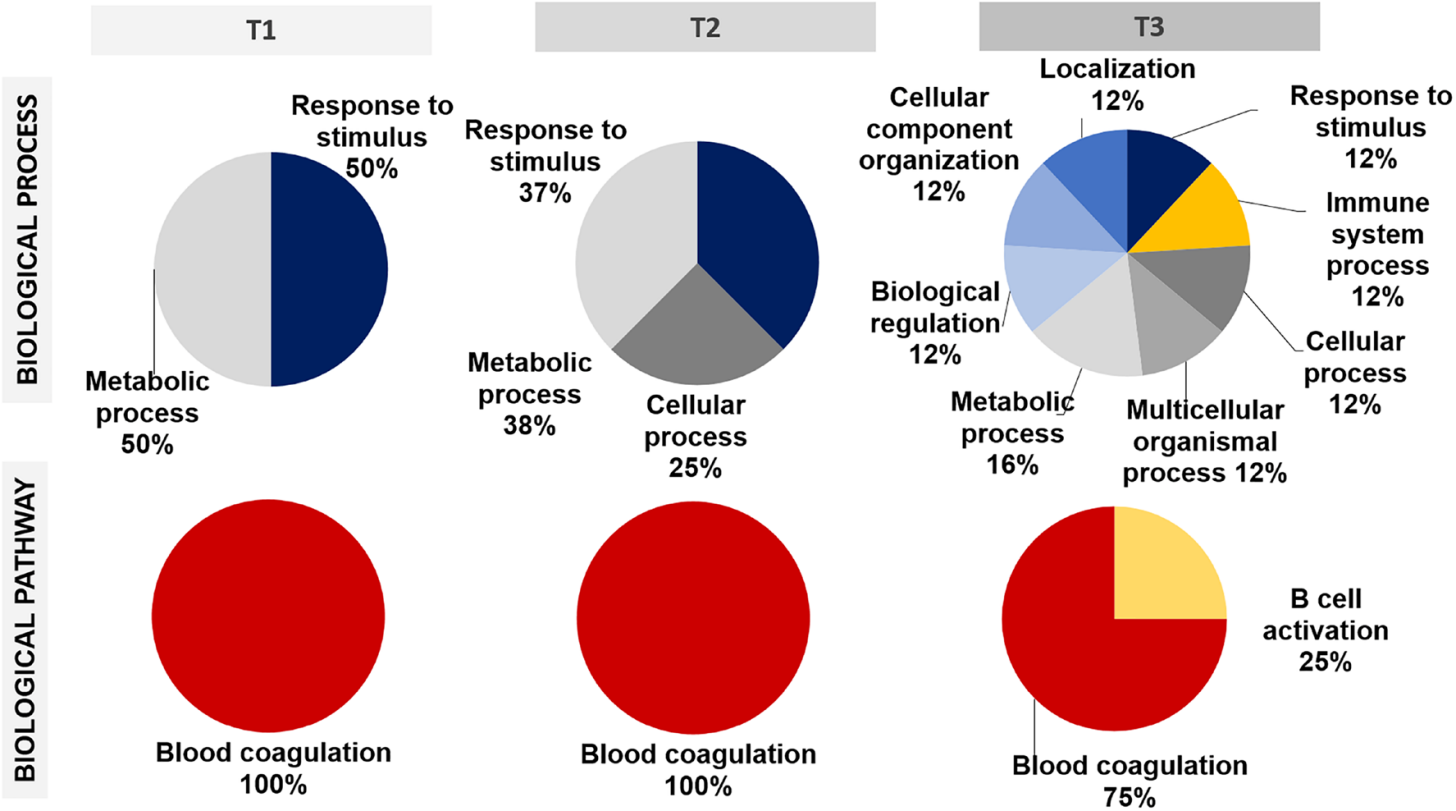
PANTHER diagrams showing biological processes and pathways associated with the proteins more abundant on Ca, relative to Control samples, *versus* contact time with human serum (T1 = 2 min, T2 = 180 min and T3 = 960 min)

PANTHER analysis revealed the involvement of the differentially adsorbed proteins in biological processes and pathways. The pie charts in Fig. 3 display the processes and pathways associated with the proteins with increased abundance on the Ca samples (in comparison with Control) versus contact time with the human serum. The proteins more adsorbed onto Ca samples after 2 min of assay were related to response to stimulus and metabolic biological process functions. After 180 min, it was also identified functions in the cellular process; and after 960 min, in localization, multicellular organismal process, biological regulation, cellular component organization and immune response. Regarding the functions associated with biological pathways, after 2 and 180 min, there was only one biological pathway associated with these proteins (blood coagulation). After 960 min, a new pathway appeared, B-cell activation (25%), in addition to the blood coagulation pathway (75%) seen before. The B-cell activation pathway is related to the immune response.

### Implant–blood contact experiments

**Fig. 4:**
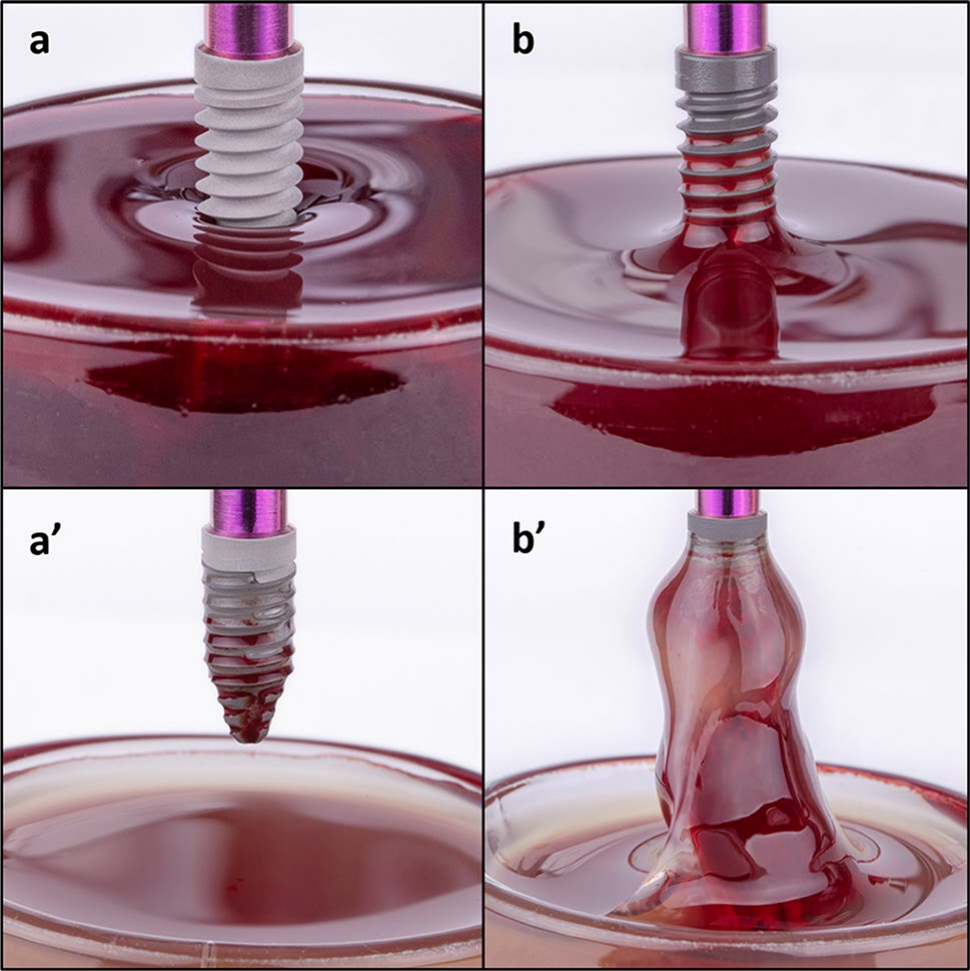
**a**, **b** Implant–blood contact and **a′**, **b′** blood coagulation after 20 min of incubation in blood for **a** control and **b** Ca surfaces

The implant surfaces were exposed to interaction with whole blood for 20 min. Upon contact with blood (Fig. 4), Control surfaces exhibited a negative meniscus: the blood level depressed around the implant contour (Fig. 4a). Blood at Ca surfaces exhibited a positive meniscus and even a capillary rise along the implant threads (Fig. 4b). After 20 min of immersion, implants were vertically pulled out. The control surface was partly wetted with blood plasma (Fig. 4a′), while Ca implants carried along a thick blood clot attached to the whole implant contour (Fig. 4b′).

### In vitro studies

#### Osteoblast cell cultures

**Fig. 5:**
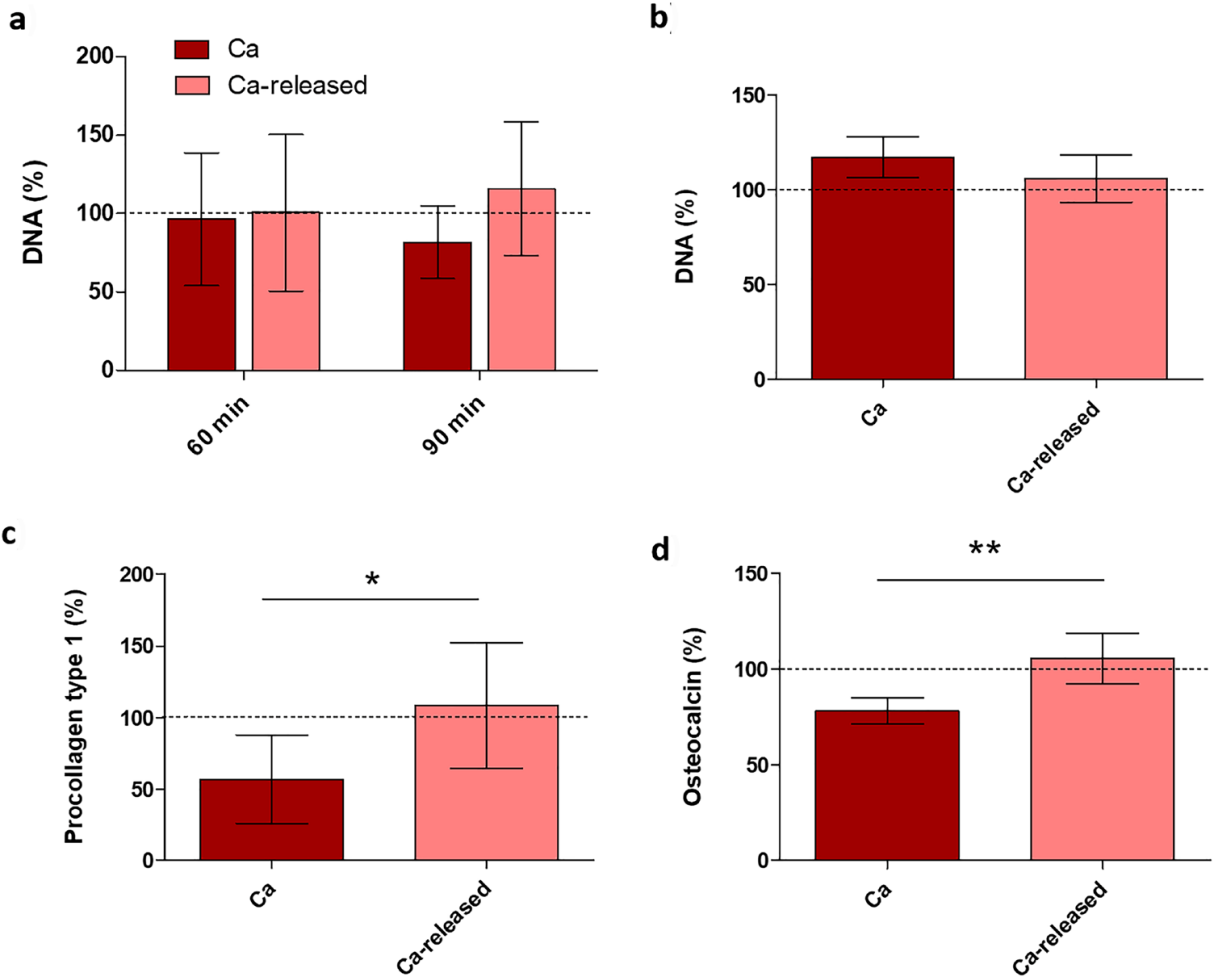
**a** Cell adhesion after 60 min and 90 min of culture and **b** cell viability after 72 h of incubation. **c** Procollagen type 1 and **d** osteocalcin synthesis at 7 days of assay. Results were calculated in percentage in relation to the Control and are represented as mean ± SE. The asterisks [*p* ≤ 0.05 (*), *p* ≤ 0.01 (**)] indicate statistically significant differences

Figure 5a presents cell adhesion measured as total DNA in the cells attached to Ca surfaces in relation to Control after 60 and 90 min. Cell viability of Ca, presented as total DNA in attached cells in percentage with respect to the Control after 72 h of culture, is shown in Fig. 5b. No significant differences were seen between cell adhesion and viability on the examined materials. The production of osteogenic procollagen type I and osteocalcin by primary human alveolar osteoblasts was measured using ELISA after 7 days of culture (Fig. 5c, d). There was an increase in osteocalcin and procollagen type I synthesis for Ca discs when the Ca is released.

#### Macrophage cell cultures: inflammatory response

**Fig. 6:**
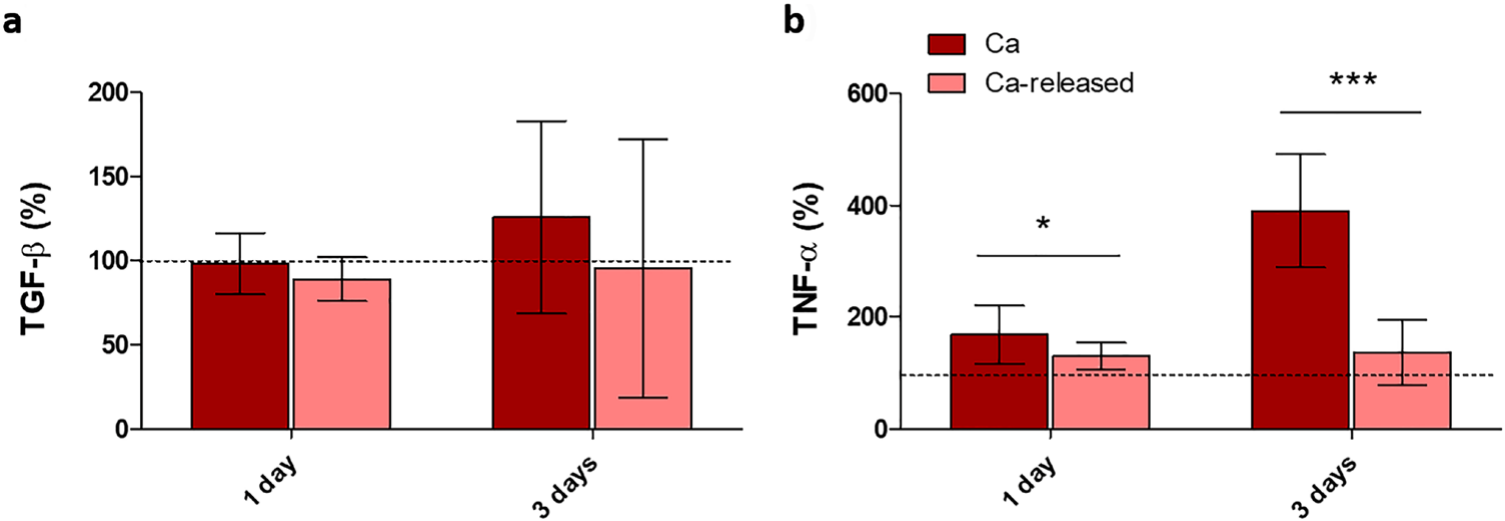
**a** TGF-β and **b** TNF-α cytokine liberation in THP-1 cells after 1 and 3 days of culture. Results were calculated in percentage in relation to the Control and are represented as mean ± SE. The asterisks [*p* ≤ 0.05 (*), *p* ≤ 0.001 (***)] indicate statistically significant differences

To evaluate how the materials affected the early (1 day) and late (3 days) inflammatory responses, the levels of TNF-α and TGF-β were measured on cell culture medium. TNF-α levels were higher in Ca samples after 1 and 3 days of exposure to the material (Fig. 6b). The secretion of TNF-α decreased in Ca-released to the levels found in Control surfaces.

## Discussion

In this study, we characterized and compared the protein layer formation onto two distinct surfaces used in dental implants: calcium modified titanium surfaces (Ca) and Ti without Ca (control), which represents a standard implant surface. The protein attachment was evaluated at several incubation periods by proteomics, obtaining the kinetics of protein adsorption onto both surfaces. Our results revealed that the Ca treatment led to a different protein layer composition with respect to the control surfaces. In addition, the distinctive protein adsorption/desorption dynamics reported onto each material could explain their effects on coagulation and inflammation.

The pro-regenerative functions of calcium has attracted the attention of researchers, and it has been consequently widely employed to develop new biomaterials for bone healing [28]. In this work, we evaluate the effect of this element when used to modify Ti surfaces. This treatment was prepared based on previous studies where it was widely characterized [19]. Figure 1 shows the initial state in which CaCl_2_ covers completely the Ti surface. The release of Ca has been reported to reach 66.5% after the first minute of contact with aqueous solutions. The remaining calcium continued to be released at a much lower rate until it became undetectable more than three months later [10]. Here, we simulated this release by washing the Ca surface prior to cell experiments.

The adsorption of proteins on the material surface significantly affects its biological outcome [29, 30]. As a result, understanding the protein layer formation onto biomaterials is of paramount importance to control their complex interaction with the biological environment [20]. Proteomic analysis revealed that 19 serum proteins were differentially adsorbed onto the Ca modified surfaces with respect to the Ti Control surface. These were mainly related to coagulatory and immune functions (Fig. 2). In fact, PANTHER results showed as the proteins preferentially adhered to calcium-enriched samples were implicated in blood coagulation pathway functions within the time-span examined (Fig. 3). The proteins associated with blood clotting were FA10, THRB and ANT3. FA10, the first member of the final common pathway, is a key protein of the coagulation cascade. The extrinsic TF-VIIa or the intrinsic (FIX-FVIIIa) tenase complexes, obtained through the extrinsic or intrinsic pathways respectively, trigger the cascade by converting FA10 (factor X) to factor Xa. Then, factor V is activated and the prothrombinase complex is formed [31]. THRB, which was also found more adsorbed onto Ca-enriched surfaces, is converted into the active protease, thrombin, by this prothrombinase complex; Ca^2+^ and phospholipids catalyse this reaction [32]. ANT3 is a serine protease inhibitor, which binds and inactivates thrombin, regulating thereby the coagulation response [33]. This regulatory protein is differentially more present onto the Ca surfaces at early stages while it becomes more present on the Control at the later stages of incubation. FA12, which can start the intrinsic pathway of coagulation [31], showed no difference in its adsorption between materials until 180 min of incubation. However, after 960 min of testing, it was identified as more adhered to the surface without Ca. The increase of FA12 with time in the reference sample compared to Ca-modified can be related to the activation of the intrinsic coagulation pathway (contact coagulation). Coagulation pathways detected are different depending on the presence of calcium (responsible for the extrinsic coagulation pathway) on the surface or not.

**Fig. 7:**
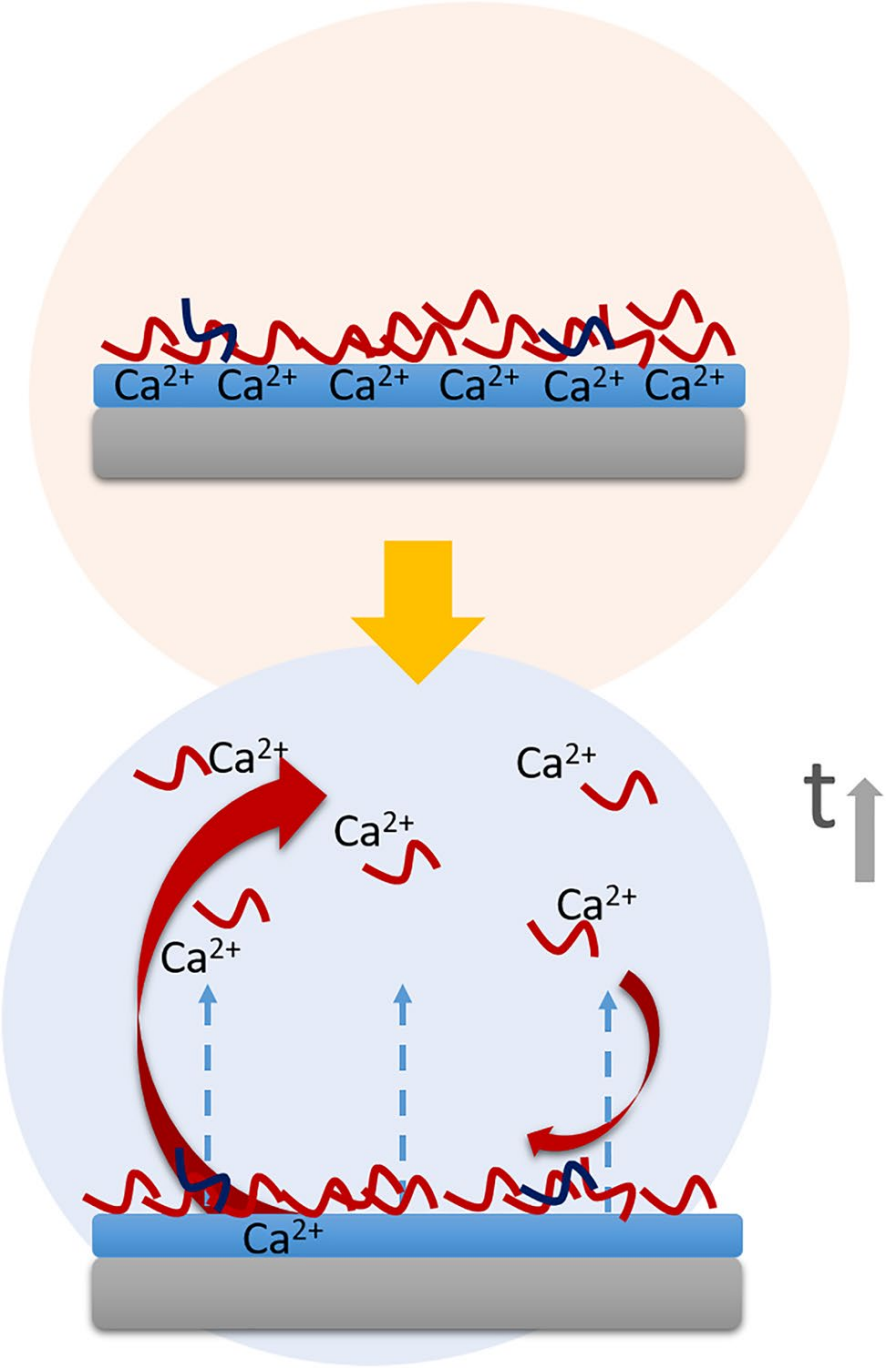
Schematic description of the changes in the first layer of proteins attached to the sample surface due to an alteration in the calcium content of the material. The release of Ca ions can lead to changes in the affinity of the proteins that are being adhered to the surface. These proteins could stop its diffusion towards the material, due to its affinity for the Ca^2+^ released in the serum. This phenomenon could promote desorption processes, reducing the presence of Ca-related proteins in the protein layer formed on the biomaterial over time

Proteins related to coagulation can increase their attachment to the Ca-treated material as a consequence of their affinity to Ca^2+^. However, the release of this element would change this interaction over time. While on the control surface these proteins increase their adsorption as the incubation period increases, on the Ca-modified surface this trend was not verified. The release of Ca^2+^ can modify the adsorption patterns, not allowing more proteins to anchor to the surface or even causing desorption phenomena as was seen in the case of FA10 (Fig. 7).

Calcium is able to modulate blood clot formation and its stability in a dose-dependent manner [37]. The adsorption kinetics of blood coagulation proteins identified in this study in the Ca samples could also condition as the clot is formed around the implant compared to the reference sample. The implant-blood contact tests showed that Ca ion-enriched surfaces promote the coagulation process compared to Control (Fig. 4). The samples with Ca attracted the formation of the clot at the implant surface instead of promoting general coagulation of the blood contained in the glass well, indicating that Ca^2+^ at these surfaces exert a bridging function between the titanium oxide and the surrounding blood. The Ca-modified implants displayed notably higher hydrophilicity than the Control surfaces. Implant hydrophilicity contributes to the stabilization of the blood clot around the entire implant surface and the recruitment and differentiation of osteogenic cells. This chain of events has been shown to translate into greater bone to implant contact and bone volume density indexes [10, 34]. Ca hygroscopic surfaces produce not only instant wetting but, via Ca ion activity, trigger a particular profile of protein adsorption at the implant surface that is tightly linked with platelet activation and fibrin formation within the coagulation process [12, 35]. The clot at the Ca implant surface represents a scaffold rich in growth factors and cell-adhesive motifs that attract and drive the development of cells down an osteogenic lineage [27]. Indeed, implant surface thrombogenicity has been related to increased rates of implant osseointegration [36, 37].

Our in vitro results did not show significant differences in the adhesion and viability of primary human alveolar osteoblasts between materials (Fig. 5a, b). Procollagen type 1 and osteocalcin synthesis were also not altered by Ca samples with respect to the control (Fig. 5c, d). However, a positive effect of Ca-enriched surfaces in adhesion, proliferation and mineralization of human foetal osteoblasts was previously reported [10]. Moreover, in vitro studies have shown that Ca^2+^ positively affects the osteogenesis of other cell types [38, 39]. Ca ion-implanted surfaces, despite demonstrating a great initial attachment of human bone marrow-derived mesenchymal stem cells, did not display an improvement of cell proliferation and differentiation at longer periods and the expression of osteoblastic type I collagen, ALP, and OC were relatively low compared with the control surface without Ca [40]. These discrepancies were related to the effect on cells of other parameters such as morphology [40]. Although, osteogenic promotion as a consequence of Ca-modified biomaterials probably has a Ca^2+^ dose-dependent effect [12] that can depend on the type of cell line used in the assay.

According to the monocyte/macrophage in vitro assays, Ca samples showed initially an increased secretion of pro-inflammatory TNF-α (Fig. 6b), which might be associated with the increased thrombogenic activity of these surfaces as these both biological systems are interconnected [41]. Silica hybrid sol–gel coatings doped with CaCl_2_ showed an enhanced TNF-α release in RAW 264.7 macrophages after 4 days of assay, which depended on the amount of Ca incorporated into the material [12]. The inflammatory potential is reduced after Ca release, as the Ca-released samples had the same TNF-α production as the Control. Therefore, Ca surfaces would not lead to a chronic inflammatory reaction. Ca-modified implants showed an improved osseointegration capacity in vivo [11].

Proteomics revealed different adsorption patterns of proteins related to immune/inflammatory functions between the Ca-enriched surfaces and the Control. The levels of adsorbed proteins related to the complement cascade as well as the number of immunoglobulins tend to decrease with time on the calcium-enriched surfaces (Fig. 2). However, the levels of C1R, IGJ, and KV121 proteins undergo a big change between 2 and 180 min of contact with the serum. This could be caused by the initial strong signal of coagulation triggered by calcium on the surface, which encompasses as well a degree of inflammatory signalling. The complement cascade proteins CO4, C1R, C1S, and CO9 were more adhered to the surfaces with Ca at very short incubation times. After 180 min, its attachment attenuated, showing no differences in adhesion with respect to the control. The adsorption of complement proteins onto biomaterials has been related to their inflammatory potential [29]. Immunoglobulins, proteins able to activate the immune reaction [42], were more present onto the Ca samples after 2 min of incubation. The increased adsorption of this type of protein, highly affected by the Ca release, is not maintained over time. In fact, IGHG1 and IGHG3 were desorbed from the Ca-surfaces after 960 min of incubation. In parallel, immunoglobulins were progressively more adsorbed onto the titanium without Ca as the incubation period increased. As a result, after 960 min of assay, KV121, IGHG3, IGHM, and IGHG1 were identified as more adsorbed onto Control than onto Ca discs. SAMP, which showed different adsorption kinetics, increases its presence over time on both surfaces, being more attached to the Ti. The role of this pentraxin on the regenerative process around biomaterials is still unclear, but it has been associated with inflammation, tissue remodelling, and coagulation [43].

In general, the increased adsorption of proteins associated with inflammation on the Ca-enriched materials at short incubation times could explain its greater inflammatory potential detected in vitro. At longer times, the changes in affinity for the surface and the desorption dynamics of this protein family would lead to a reduction in inflammation as observed on the Ca-released surfaces.

In the light of recent findings, it should not be forgotten the crosstalk between coagulation and the complement system, which can be viewed as partners in the inflammatory responses of a living organism to the various disturbances in its homeostasis. It is well established that the complement system is activated during blood clotting, augmenting the thrombogenic properties of blood through the inhibition of anticoagulation mechanisms. Multiple regulatory loops linking the two systems are simultaneously activated to synchronise an effective response [21]. The present study helps to pave the way for the understanding of how these key systems in the regeneration process interact with Ca-enriched materials.

## Conclusion

The aim of this study was to characterize Ca-enriched titanium surfaces and how it modulates protein adsorption through time and the in vitro cell responses of osteoblasts and macrophages. The presence of calcium on the surface did not change its osteogenic potential in vitro in comparison with the control samples, while increased coagulation and inflammation in the initial stage of the regeneration process. The proteomic characterisation revealed calcium-dependent changes in the adsorption patterns of serum proteins. Calcium-enriched materials displayed a higher affinity for proteins related to coagulation, suggesting an increase in the coagulatory potential of these materials. With prolonged contact with the serum, the protein layer on the materials changed; the levels of the proteins associated with coagulation and inflammation diminished with time. These results improve our understanding of the material-biological interface interaction, showing how the protein layers change throughout time and ultimately modulate cell responses. In the case of Ca-based materials, this is of particular importance, considering the relevance of this ion to the coagulation and overall cell responses.

## Supplementary Information

Below is the link to the electronic supplementary material.

Supplementary file1 (PDF 40 KB)
