# Supplementary material for: Protein adsorption/desorption dynamics on Ca-enriched titanium surfaces: biological implications
Source: J Biol Inorg Chem. 2021 Aug 27;26(6):715–26. doi: 10.1007/s00775-021-01886-4 (PMC8437886; doi:10.1007/s00775-021-01886-4)
Supplement: Supplementary file 1 — Supplementary file1 (PDF 40 KB) [file 775_2021_1886_MOESM1_ESM.pdf]

Online Resource 1 Progenesis analysis of proteins differentially adsorbed onto Ca samples in comparison with the Control samples for 3 different durations of contact with the serum (T1 = 2 min, T2 = 180 min and T3 = 960 min). The differentially attached proteins are highlighted in yellow. Proteins detected with ANOVA, with $p < 0.05$, and a ratio of more than 1.5 in either direction (the increase is shown in red and decrease in green), were considered significantly different (*)

				T1 Ca/Control		T2 Ca/Control		T3 Ca/Control	
**Description**	**Accession**	**Peptide count**	**Confidence score**	**p value**	**Ratio**	**p value**	**Ratio**	**p value**	**Ratio**
**Coagulation factor X**	FA10_HUMA	3	106.79	8.04E-05	41.24*	1.77E-06	130.24*	2.71E-04	19.73*
**Complement C1s**	C1S_HUMAN	4	210.53	4.68E-02	2.98*	1.40E-01	2.2	6.56E-01	1.09
**Complement C4-A**	CO4A_HUMA	21	1138.33	8.77E-02	2.78	2.23E-03	2.96*	1.56E-01	1.31
**Prothrombin**	THRB_HUMA	12	586.21	3.19E-05	2.52*	2.14E-03	3.02*	1.83E-01	1.28
**Complement component C9**	CO9_HUMAN	3	172.2	1.15E-02	2.52*	8.69E-01	0.93	9.71E-01	0.86
**Antithrombin-III**	ANT3_HUMA	6	348.96	3.17E-02	2.23*	1.09E-01	1.7	8.52E-03	0.59*
**Protein AMBP**	AMBP_HUM	3	136.32	6.16E-03	1.92*	3.00E-01	0.71	6.31E-01	1.12
**Ig kappa chain V-III region**	KV302_HUM	3	174.94	1.09E-02	1.66*	4.81E-01	0.62	1.85E-01	0.45
**Ceruloplasmin**	CERU_HUMA	3	145.06	1.78E-01	1.63	4.42E-01	0.81	1.73E-03	0.32*
**Ig kappa chain V-I region Ni**	KV121_HUM	2	99.47	3.19E-01	1.59	9.88E-02	2.04	4.03E-02	0.54*
**Ig gamma-3 chain C region**	IGHG3_HUM	14	644.61	5.03E-01	1.41	3.19E-01	1.42	3.46E-03	0.49*
**Ig mu chain C region**	IGHM_HUMA	7	362.7	3.50E-01	1.39	4.99E-01	1.12	3.93E-02	0.55*
**Complement C1r**	C1R_HUMAN	3	141.38	5.81E-01	1.28	1.78E-02	3.34*	9.71E-01	1.07
**Coagulation factor XII**	FA12_HUMA	4	121.84	7.23E-01	1.08	4.62E-01	0.37	9.81E-03	0.31*
**Selenoprotein P**	SEPP1_HUMA	2	100.05	9.17E-01	1.07	3.71E-02	4.96*	7.36E-01	0.96
**Ig gamma-1 chain C region**	IGHG1_HUM	12	647.42	7.77E-01	0.96	4.36E-01	1.13	4.61E-02	0.65*
**Apolipoprotein B-100**	APOB_HUMA	8	324.46	9.54E-01	0.71	5.83E-02	0.12	2.61E-02	0.23*
**Immunoglobulin J chain**	IGJ_HUMAN	3	123.71	5.60E-01	0.71	4.47E-02	1.83*	5.86E-01	0.82
**Serum amyloid P-component**	SAMP_HUMA	4	219.87	8.40E-01	0.43	4.27E-03	0.08*	2.42E-02	0.2*
